# How Experiences Differ

**Published:** 1887-07

**Authors:** 


### HOMŒOPATHIC PHYSICIAN,

A MONTHLY JOURNAL OF MEDICAL SCIENCE.

"If our school ever gives up the strict inductive method of Hahnemann, we are lost, and deserve only to be mentioned as a caricature in the history of medicine."—CONSTANTINE HERING.

Vol. VII. JULY, 1887. No. 7.

### EDITORIAL NOTES.

HOW EXPERIENCES DIFFER.—In a recent article upon a question of diet, we find this comment upon the differences of opinion and hence (among the allopaths) of practice. "Here, as in other articles and discussions one hears in societies and conversations, it is strikingly remarkable how few observers see alike, how few thinkers think alike, and how few experiments yield alike in the hands of different experimenters."

It is hardly necessary to state that the above quotation is from the pen of an allopath; one versed in homœopathic philosophy would easily and surely recognize the ring of doubt which sounds through the remark. With them all is in doubt, nothing is certain—all is to be settled by experience, nothing by law.

How different are the discussions of homœopathic bodies, how general is the agreement, and how unanimous is the experience of those who practice by certain law. And this agreement in theory and in practice is not obtained solely from physicians living in the same country; it is fully evidenced by the experience of homœopaths throughout the world. The homœopathic law has been found universally active in America or in Europe, in cold or in hot climates. It never fails, though its practitioners do fail.

